# *FADS1* Genetic Variant and Omega-3 Supplementation Are Associated with Changes in Fatty Acid Composition in Red Blood Cells of Subjects with Obesity

**DOI:** 10.3390/nu16203522

**Published:** 2024-10-17

**Authors:** Samantha Desireé Reyes-Pérez, Karina González-Becerra, Elisa Barrón-Cabrera, José Francisco Muñoz-Valle, Juan Armendáriz-Borunda, Erika Martínez-López

**Affiliations:** 1 Doctorado en Ciencias en Biología Molecular en Medicina, Centro Universitario de Ciencias de la Salud, Universidad de Guadalajara, Sierra Mojada 950, Guadalajara 44340, Jalisco, Mexico; samantha.reyes7868@alumnos.udg.mx; 2 Instituto de Nutrigenética y Nutrigenómica Traslacional, Centro Universitario de Ciencias de la Salud, Universidad de Guadalajara, Sierra Mojada 950, Guadalajara 44340, Jalisco, Mexico; 3 Instituto de Investigación en Genética Molecular, Departamento de Ciencias Médicas y de la Vida, Centro Universitario de la Ciénega, Universidad de Guadalajara, Av. Universidad 1115, Ocotlán 47810, Jalisco, Mexico; karina.gbecerra@academicos.udg.mx; 4 Posgrado en Ciencias de la Nutrición y Alimentos Medicinales, Facultad de Ciencias de la Nutrición y Gastronomía, Universidad Autónoma Sinaloa, Av. Cedros y Calle Sauces S/N, Culiacán 80010, Sinaloa, Mexico; elisabarron@uas.edu.mx; 5 Instituto de Ciencias Biomédicas, Departamento de Clínicas Médicas, Centro Universitario de Ciencias de la Salud, Universidad de Guadalajara, Sierra Mojada 950, Guadalajara 44340, Jalisco, Mexico; drjosefranciscomv@cucs.udg.mx; 6 Instituto de Biología Molecular en Medicina y Terapia Génica, Centro Universitario de Ciencias de la Salud, Universidad de Guadalajara, Sierra Mojada 950, Guadalajara 44340, Jalisco, Mexico; armdbo@gmail.com; 7 Escuela de Medicina y Ciencias de la Salud, Tecnologico de Monterrey, Campus Guadalajara, Av. Gral Ramón Corona No. 2514, Colonia Nuevo México, Zapopan 45201, Jalisco, Mexico

**Keywords:** obesity, omega 3, *FADS1*, inflammation, genetic variant

## Abstract

Introduction: Obesity is characterized by low-grade chronic inflammation, which can be modulated by lipid mediators derived from omega-3 (*n*-3) polyunsaturated fatty acids (PUFA). Obesity is a multifactorial disease, where genetic and environmental factors strongly interact to increase its development. In this context, the *FADS1* gene encodes the delta-5 desaturase protein, which catalyzes the desaturation of PUFA. The rs174547 genetic variant of *FADS1* has been associated with alterations in lipid metabolism, particularly with decreases in eicosapentaenoic acid (EPA) and arachidonic acid (AA) concentrations. Objective: To analyze the effect of an *n*-3-supplemented diet on the fatty acid profile and composition in red blood cells (RBCs) of obese subjects carrying the rs174547 variant of the *FADS1* gene. Methodology: Seventy-six subjects with obesity were divided into two groups: omega-3 (1.5 g of *n*-3/day) and placebo (1.5 g of sunflower oil/day). The dietary intervention consisted of a four-month follow-up. Anthropometric, biochemical, and dietary variables were evaluated monthly. The total fatty acid profile in RBC was determined using gas chromatography. The rs174547 variant was analyzed through allelic discrimination. Results: The *n*-3 index (O3I) increased at the end of the intervention in both groups. Subjects carrying the CC genotype showed significant differences (minor increase) in *n*-6, *n*-3, total PUFA, EPA, DHA, and the O3I in RBCs compared to TT genotype carriers in the *n*-3 group. Conclusions: The diet supplemented with EPA and DHA is ideal for providing the direct products that bypass the synthesis step affected by the *FADS1* rs174547 variant in subjects carrying the CC genotype. The O3I confirmed an increase in *n*-3 fatty acids in RBCs at the end of the intervention.

## 1. Introduction

Obesity is one of the most prevalent chronic diseases in the world, defined as an excessive accumulation of body fat that can be harmful to health. It is responsible for 2.8 million deaths per year and represents a risk factor for the development of other pathologies, such as high blood pressure and metabolic syndrome, among others [1].

Obesity is a multifactorial disease in which social, genetic, and environmental factors interact to contribute to its development. It has been proposed that these factors are interrelated and have a significant influence on the progression of the disease. Recently, it has been described that an unhealthy lifestyle, along with inappropriate habits and behaviors, represent risk elements that can promote an obesogenic environment [2].

The presence of low-grade chronic inflammation is a specific condition in obesity, which actively persists in the disease and exacerbates various metabolic alterations [3]. This inflammation, also known as lipoinflammation, can be modulated by lipid mediators derived from polyunsaturated fatty acids (PUFA). Omega-6 (*n*-6) and omega-3 (*n*-3) PUFAs are the main precursors of molecules involved in the inflammatory and anti-inflammatory processes, respectively. The primary molecules derived from *n*-6 PUFAs are linoleic acid (LA) and arachidonic acid (AA). The main molecules derived from *n*-3 PUFAs are alpha-linolenic acid (ALA), eicosapentaenoic acid (EPA), and docosahexaenoic acid (DHA), which have been attributed anti-inflammatory functions due to their ability to suppress the expression of pro-inflammatory cytokines by modulating transcription factors, primarily peroxisome proliferator-activated receptor gamma (PPARγ) [4].

Currently, Western dietary patterns exhibit a deficient intake of *n*-3 PUFAs and an excess consumption of *n*-6 PUFAs, which favors an imbalance in the *n*-6:*n*-3 ratio, promoting the development of pro-inflammatory conditions [5]. Furthermore, the direct impact of PUFA intake on the human body can be assessed through the analysis of RBCs. Primarily, the composition of the fatty acid profile in RBCs represents an appropriate biomarker to evaluate dietary intake and fatty acid metabolism in relation to the inflammatory processes characteristic of obesity [6].

Recent advances in scientific research have prioritized the analysis of molecules involved in inflammatory processes. The fatty acid desaturase (FADS) family is associated with the biosynthesis and metabolism of PUFAs. In particular, the *FADS1* gene has been widely studied. It encodes the delta-5 desaturase enzyme (D5D), which is involved in the desaturation of PUFAs. However, this gene has also been studied for the presence of several genetic variants. One of the genetic variants with the most scientific evidence is rs174547, which is related to decreased enzymatic activity of the D5D protein. Consequently, it has been associated with alterations in the products of PUFAs, mainly EPA and DHA. Therefore, the aim of this study was to analyze the effect of an *n*-3-supplemented diet on the fatty acid profile and composition in RBCs of obese subjects carrying the *FADS1* rs174547 variant.

## 2. Methods

### 2.1. Study Population

The study enrolled a total of 314 subjects from the Metropolitan Zone of Guadalajara as the reference population. The participants were patients with obesity who originally participated in a randomized clinical trial analyzing the effect of *n*-3 PUFA supplementation on the total fatty acid profile and inflammatory markers (ClinicalTrials.gov, NCT04901052). From this cohort, 82 individuals were recruited to receive dietary treatment. However, the entire sample was used as the reference population to generate the first report on the frequency of this variant in the Mexican population. Additionally, allelic and genotypic frequencies were compared to ensure that a similar allelic distribution was maintained in the smaller sample.

The study was carried out in January 2019 and included 82 subjects with obesity (men and women aged 30 to 50 years) who were randomly assigned to 2 groups: placebo and omega-3. During the four-month intervention, six participants dropped out of the study. Consequently, a total of 76 individuals completed the study, with 38 subjects in each group. The exclusion criteria included pregnancy, breastfeeding, and consumption of nutritional supplements. Additionally, subjects with a history of medication use, including hypolipemic, hypoglycemic, and non-steroidal anti-inflammatory drugs for at least one year, or those with metabolic diseases (DM2, nonalcoholic steatohepatitis (NASH), nonalcoholic fatty liver disease (NAFLD), cardiovascular diseases, or any type of cancer) were excluded. Moreover, an initial clinical and anthropometric pre-evaluation was conducted by nutritionists, and subjects who met the inclusion criteria and agreed to participate were required to sign an informed consent. This study was approved by the Ethics and Biosafety Committee of the Centro Universitario de Ciencias de la Salud at the Universidad de Guadalajara, Jalisco, Mexico (Registration CI-01219), and adhered to the WMA Declaration of Helsinki (1964), as amended by the 64th WMA General Assembly in Fortaleza, Brazil, 2013 [7].

### 2.2. Dietary Intervention

Participants were initially interviewed for demographic, family history, pathological, and lifestyle information using a standardized questionnaire. Similarly, a 24 h dietary recall and a three-day food diary were used (two weekdays and one weekend day). Participants were instructed to recall specific details regarding the quantity and type of food consumed, and during the interview, the nutritionist used food-scale models from Nasco^®^ (Fort Atkinson, WI, USA) to improve portion size accuracy. Nutritional data were analyzed using Nutritionist Pro^™^ software 8.1 (Axxya Systems, Woodinville, WA, USA).

Both groups received nutritional counseling over a period of four months. The nutritional intervention consisted of a 20% reduction in total energy expenditure, estimated using the Mifflin formula (based on baseline weight), with a macronutrient distribution of 50% carbohydrates, 30% lipids (<7% saturated fatty acids (SFA), 10% monounsaturated fatty acids (MUFA), 10% polyunsaturated fatty acids (PUFA), and an *n*-6:*n*-3 ratio of 5:1), and 20% protein. Additionally, the placebo group received two capsules per day containing 1.5 g of sunflower oil, while the intervention group (omega-3 group) received two capsules per day of *n*-3 containing 1.5 g of *n*-3 (1000 mg EPA and 500 mg DHA). The *n*-3 capsules were obtained from the same batch, and a toxicity analysis was performed to verify their safety. Finally, during the follow-up, educational materials were provided to all subjects to support compliance and adherence to the dietary plan.

### 2.3. Adherence Evaluation

Dietary adherence was assessed using a multifaceted approach. First, self-reported data from participants were utilized. Additionally, a thorough analysis of their three-day dietary records was conducted, focusing on the percentage of macronutrient adequacy and the *n*-6:*n*-3 ratio. In this sense, an overall adherence percentage was calculated, incorporating both the self-reported values and the dietary analysis, with particular emphasis on compliance with the *n*-6:*n*-3 ratio. Finally, adherence to taking the capsules was documented by the return of their capsule bottle.

### 2.4. Biochemical Measurements

Venous blood samples were collected after 12 h of fasting. Serum biochemical evaluations were performed using dry chemistry with the Vitros 350 Analyzer (Ortho-Clinical Diagnostics, Johnson and Johnson Services, Inc., Rochester, NY, USA). The biochemical parameters included glucose and lipid profiles, including triglycerides (TG), total cholesterol (CT), and high-density lipoprotein cholesterol (HDL-c). Low-density lipoprotein cholesterol (LDL-c) was calculated using Friedewald’s formula, except when triglyceride levels were higher than 400 mg/dL, and very low-density lipoprotein cholesterol (VLDL-c) was estimated by dividing total triglycerides by 5. Insulin levels were measured using an ELISA Kit (International Diagnostics, Guadalajara, Jalisco, Mexico. S.A de C.V) following the supplier’s instructions. All biochemical determinations were analyzed monthly and up to the fourth month post-intervention.

### 2.5. Total Fatty Acid Profile of the Erythrocytes

The quantification of the fatty acid profile in RBCs was performed using gas chromatography. A total of 37 fatty acids were measured, as detailed in the Supplementary Materials (Table S1). This procedure involved adding 250 µL of erythrocytes to a hatch tube, followed by the addition of 6 mL of Folch solution (chloroform, 2:1 *v*/*v*) and 10 µL of the antioxidant butylated hydroxytoluene (0.01% BHT). The samples were sonicated in an ice bath for 20 min and stored at −20 °C for 24 h. Subsequently, two cycles of sonication and homogenization were performed (Christie’s technique, 2003 [8]). For direct derivatization of total lipids, samples were evaporated using gaseous nitrogen, and 2.5 mL of methanol and hydrochloric acid solution (95:5 *v*/*v*) was added and incubated in a water bath at 85 °C for 2.5 h. After cooling the samples, 1 mL of hexane was added, homogenized, and centrifuged. The hexane–lipidic phase was washed and stored at −20 °C (Morrison and Boyd, 1990 [9]). Once the aqueous phase was frozen, the hexane–lipid phase was transferred to an amber vial, and 60 µL was prepared for injection into the gas chromatograph. The analysis was performed using an Agilent Technologies gas chromatograph (GC) (Agilent Technologies, Santa Clara, CA, USA, 6850 network GS system) coupled with an injector (Agilent Technologies, 7083 Series) with a column for fatty acids (Durabond, DB-23 Agilent Technologies), and a flame ionization detector, with helium as the carrier gas, programmed for a temperature ramp of 30 °C/min from 110 °C to 250 °C over 56.42 min. Fatty acids were identified and quantified based on the retention times compared with a standard chromatogram (Marinol^®^), with the percentage of fatty acids calculated using the following formula: Fatty acids (%) = A/B × 100, where A represents the total area of the fatty acid in the sample and B the sum of all fatty acid areas. The results are expressed as a percentage relative to 100% of the sample.

The O3I was calculated as the proportion of EPA (20:5 *n*-3) and DHA (22:6 *n*-3) relative to the total fatty acids in RBC.

### 2.6. DNA Extraction and Genotyping

Peripheral blood was collected in EDTA tubes to extract genomic DNA (gDNA) from peripheral leukocytes using the Roche High Pure PCR Template Preparation Kit, with 200 µL of blood. Nucleic acids were quantified by spectrophotometry using a ThermoFisher Scientific Nanodrop 2000c UV instrument (Thermo Scientific, Waltham, MA, USA).

The genotyping assay was carried out using allelic discrimination tests with TaqMan^®^. The rs174547 genetic variant (assay number: C___2292336_10, catalog number 4351379, Applied Biosystems, Foster City, CA, USA) was determined using a real-time PCR system. Subsequently, the PCR reaction and fluorescence measurement were performed using a real-time LightCycler 96 (Roche, Basel, Switzerland) system according to the supplier’s specifications. The assay was verified using positive controls of DNA samples corresponding to the three possible genotypes, as well as negative controls, in each 96-well plate assay.

### 2.7. Statistical Analysis

Data were analyzed using SPSS v.20 software (IBM, Chicago, IL, USA). The Shapiro–Wilk test was used to evaluate data distribution. Quantitative variables were analyzed using the Student’s *t*-test and the Mann–Whitney U test, depending on the normality results.

To evaluate changes from baseline to final, the paired Student’s *t*-test or the Wilcoxon test was used, depending on normality, and changes in dietary variables were logarithmically transformed. The comparison of means between the two treatment groups was performed using the unpaired Student’s *t*-test according to normality. The Hardy–Weinberg equilibrium model was used to analyze population equilibrium. A *p*-value < 0.05 was considered statistically significant.

## 3. Results

### 3.1. Comparison of Allelic and Genotypic Frequencies between the Intervention Group and the Reference Population

Table 1 presents the genotype and allelic frequencies reported by the 1000 Genomes Project, the Mexican reference population, and the study groups. The reported frequencies were similar across the different populations, and no statistically significant differences were found between them (*p* = 0.71). According to the Hardy–Weinberg equilibrium model, the variant of interest was in equilibrium (*p* = 0.88).

### 3.2. Description of the Sociodemographic, Anthropometric, Biochemical, and Nutritional Characteristics of the Study Population

From this point forward, the data correspond to participants who received the nutritional intervention with *n*-3 supplementation or the placebo during a 4-month follow-up, in which 76 subjects with obesity were enrolled.

No significant differences were found between the groups in demographic, dietary, and biochemical variables at baseline, highlighting appropriate randomization (Table 2 and Table 3). The average age of the placebo group was 38.1 ± 11.2 years, while the omega-3 group had an average age of 37.1 ± 5.8 years. Similarly, the proportion of women was higher at 68.4%, while the proportion of men was 31.6% in both groups.

### 3.3. Changes in Dietary Variables by Study Group

Table 4 presents the changes in dietary variables from baseline to the end of the intervention. The placebo group showed a significant increase in EPA 20:5 (*p* = 0.008) and DHA 22:6 (*p* = 0.032). In the omega-3 group, there was a significant decrease in energy and carbohydrate consumption (*p* = 0.023 and *p* = 0.038, respectively).

Additionally, adherence to dietary recommendations showed a progressive decrease toward the end of the intervention. In this regard, the placebo group showed higher adherence to dietary recommendations compared to the omega-3 group.

### 3.4. Relationship Between the FADS1 rs174547 Variant and the Fatty Acid Profile in RBCs in Response to an Intervention

Additionally, the O3I was calculated, referring to the percentage of EPA and DHA in RBCs. Table 5 presents the changes in the incorporation of EPA and DHA after the intervention, showing a significant increase in both study groups but no significant differences between the groups at the end of the intervention.

Table 6 presents the multiple intragroup and intergroup comparisons of the lipid profile in RBC by genotype (CC and TT homozygotes). Regarding polyunsaturated fatty acids, the CC genotype in the placebo group showed a significant post-intervention increase in polyunsaturated fatty acids AA, DHA, and O3I. Similarly, the TT genotype in the placebo group exhibited a significant post-intervention increase in *n*-3, EPA, DHA, and O3I. When comparing ∆CC vs. ∆TT within the placebo group, significant changes were observed in EPA, DHA, and O3I, with a smaller increase in the CC genotype.

On the other hand, the CC genotype in the omega-3 group showed significant post-intervention changes in total *n*-6, LA, and total *n*-3. For the TT genotype in the omega-3 group, significant changes were observed in most fatty acids, with a particular increase in total PUFA, *n*-6, LA, AA, *n*-3, EPA, DPA, DHA, and O3I. Subsequently, when comparing ∆CC vs. ∆TT within the omega-3 group, changes were also observed in most fatty acids, particularly in PUFA, *n*-6, LA, AA, *n*-3, and EPA, with a smaller increase in the CC genotype. Finally, intergroup comparisons (placebo vs. omega-3) for the CC genotype showed significant changes in *n*-6, AA, EPA, and DHA. Meanwhile, the intergroup comparison (placebo vs. omega-3) for the TT genotype revealed significant changes mainly in PUFA, *n*-6, LA, AA, and DPA.

## 4. Discussion

The present study reported that subjects carrying the CC genotype of the *FADS1* rs174547 variant showed a smaller increase in *n*-6, *n*-3, total PUFA, and EPA percentages in RBCs compared to TT genotype carriers, in subjects with obesity supplemented with *n*-3.

Although the molecular mechanism of the single-nucleotide variant (SNV) of interest has not been fully elucidated, several hypotheses have been proposed, two of which are widely accepted. The first suggests that two variants, rs174545 and rs174546, are in linkage disequilibrium with the rs174547 variant. Several in vitro studies have conducted functional tests and identified that the rs174546 SNV had a greater effect on desaturase activity, as it generated a C>T transition in the 3′UTR of the *FADS1* gene, promoting the binding of four possible miRNAs (miR-149-5p, miR-942-5p, miR-1237-3p, and miR-484). miR-149-5p showed the greatest association, as it bound to the gene and promoted an allele-specific illegitimate binding site, leading to post-transcriptional inhibition of *FADS1* gene expression and, consequently, reducing D5D enzymatic activity by 60% [10].

The second proposed mechanism involves another variant, rs174557, which is in linkage disequilibrium with rs174547 and is located in intron 2 of the *FADS1* gene. In this context, the variant played an important role in the transcription factor binding site for sterol-regulatory element-binding protein (SREBP1c), which requires interaction with the SP1 protein to form an activator complex for the gene. When the variant was present, a C>A transversion occurred, leading to higher affinity for the transcription factor PATZ1 (BTB-POZ Domain Zinc Finger Transcription Factor), which acted as a suppressor by blocking the activator complex from binding, as it competed for the same site. Therefore, the effect of the rs174547 variant could be linked to its interaction with these variants in linkage disequilibrium, as it has the strongest evidence of reducing desaturase activity [11,12].

Previous studies analyzing the effect of the rs174547 variant on PUFA percentages have primarily been conducted in East Asian countries. Therefore, this research provided relevant information regarding the frequencies of this variant in the Mexican population.

In the context of the intervention study, the dietary data indicated that the study population had an imbalance in the nutrient consumption and *n*-6:*n*-3 ratio, which increases the risk of developing inflammatory diseases. These results are consistent with those reported in the Mexican population by Campos-Perez et al., who analyzed the average macronutrient consumption of subjects, finding a high intake of SFA, total cholesterol, and trans-fatty acids, as well as a deficient intake of PUFA [13].

It has been proposed that an imbalance in macronutrient distribution and types of fatty acids increases the risk of developing metabolic alterations [14,15]. Similarly, this lipid profile leads to increased activation of Toll-like receptors (TLRs), greater macrophage recruitment, and the release of molecules, such as TNFα and IL-6 [16], resulting in deregulation of adipose tissue, peripheral adipocytes’ death, and alterations in insulin signal transduction [17].

In relation to the nutritional intervention by study group, the results of the present study showed significant changes by the end of the intervention. An increase in EPA and DHA consumption was recorded in the placebo group, while only a decrease in energy and carbohydrate consumption was observed in the *n*-3-supplemented group. The increase in EPA and DHA corresponded to dietary intake, calculated in the meal plans provided to participants, with a focus on foods rich in omega-3. These results reflected greater adherence to dietary recommendations in the placebo group. Our data partially differ from those reported by Cormier et al., who evaluated the effect of *n*-3 fatty acid supplementation in relation to the SNV rs174546, which, as mentioned, is in linkage disequilibrium with rs174547. Their results after supplementation showed a significant decrease in energy consumption and SFA, along with an increase in PUFA consumption. The differences between the results may be due to Cormier’s study having a larger sample size (208 subjects), a higher dose (3 g of *n*-3: 1.9 g of EPA and 1.1 g of DHA), and a shorter intervention period (six weeks). Additionally, the differences may primarily be due to the level of dietary adherence; however, in the aforementioned study, adherence was not evaluated [18].

Numerous studies have evaluated the effects of *n*-3 fatty acids in the prevention and treatment of metabolic diseases by analyzing the fatty acid composition in blood, which reflects exogenous fatty acid consumption. Initially, the lipid composition of the cell membrane can be modified by dietary fat intake, thereby altering key cell functions, such as transport, transmembrane receptor activity, production of mediators, and signaling. In our study, the fatty acid composition in RBCs was analyzed as a suitable biomarker that reflects chronic intake over the previous 90 to 120 days and is considered superior to serum or plasma determinations [19,20]. This analysis of fatty acid composition in RBCs enabled sub-analyses through the calculation of the O3I, which is derived from the sum of EPA and DHA relative to the total fatty acid content in RBCs. The changes observed at the end of the intervention showed a significant increase in this parameter within each of the intervention groups. This result provides a method for evaluating adherence as a key measure of EPA and DHA intake in the study groups. We considered this one of the most significant findings, as both groups achieved a significant increase in the percentage of EPA and DHA incorporation in response to the nutritional intervention. In fact, the omega-3 group reached an optimal percentage, as scientific evidence suggests that an O3I ≥ 8% is associated with a reduced risk of chronic diseases, particularly cardiovascular disease [21].

Conversely, no significant differences were observed between the groups at the end of the intervention. Baseline values appeared to be a determining factor in this outcome, as we observed a greater increase in the placebo group, which initially recorded a lower O3I. This finding contrasts with previous studies that reported a more substantial increase in individuals with lower baseline O3I as a result of intervention. Meanwhile, individuals with a higher O3I showed a lower response to treatment, suggesting that those with elevated baseline EPA + DHA levels incorporate additional EPA + DHA at a slower rate compared to those with lower baseline levels [22].

Regarding the relationship between the SNP rs174547 of *FADS1* and fatty acid composition in RBCs in response to an *n*-3-supplemented diet, both groups exhibited a smaller increase in the percentages of *n*-6 and *n*-3 PUFAs in the CC genotype compared to the TT genotype. Notably, the placebo group showed significant differences in the increase of the O3I, where the TT genotype (non-risk genotype) doubled the increase in the O3I compared to carriers of the CC genotype (risk genotype). Meanwhile, although the omega-3 group showed a lower increase in the percentage of incorporated EPA, the omega-3 group did not reach statistical significance, instead showing a trend of borderline significance (*p* = 0.064).

These data are partially consistent with those reported by Muzsik and colleagues, who observed statistically significant differences between genotypes in the concentrations of ALA, DGLA, *n*-6, and PUFAs (approximately 70%, 40%, 35%, and 35%, respectively), with lower incorporation in the CC + CT genotype compared to the TT genotype receiving the Central European diet intervention. Both studies found that, following nutritional intervention, differences were observed in the percentage of *n*-3 and *n*-6 PUFAs. These changes suggest that PUFA metabolism may be limited by diet and genotype, similar to our findings, which indicated associations between *FADS* genotypes and fatty acid concentrations [23].

Several studies have shown that carriers of the minor C allele had a higher proportion of substrates and a lower increase in products, demonstrating reduced D5D and D6D activity in the synthetic pathway. Specifically, a study conducted by Merino and colleagues examined the regulation of desaturase activity due to genetic variation in the *FADS1* gene within two young populations: Caucasians and Asians. This research also assessed whether desaturase activity was reflected in the *n*-3 and *n*-6 lipid profiles. Among the cluster of variants analyzed, the most significant association was observed with the rs174547 variant in both populations, where carriers of the C allele exhibited lower desaturase activity compared to those with the T allele. This finding serves as a reference point, indicating that the rs174547 variant was dominant within the *FADS* gene cluster, with a more pronounced and contrasting effect observed among homozygous genotypes [24]. Although our results were not entirely consistent with these findings, a meta-analysis related to the *FADS1* rs174547 variant indicated that the composition of precursor fatty acids and products differed when there was a change in the *FADS1* SNP. The associated effects of this variant on fatty acid concentrations may also be modified by dietary intake [25,26].

The analysis of the relationship between the rs174547 variant of the *FADS1* gene and the percentages of fatty acids in RBCs confirmed the effect of the CC genotype on the alteration of PUFA products. Minor increases in fatty acid levels were observed in CC genotype carriers across both groups. Additionally, the increase in the O3I at the end of the intervention supports the hypothesis that consuming at least 1 g of *n*-3 per day increases the O3I by 2%, underscoring the importance of the dietary intervention in addressing low-grade chronic inflammation in obesity [27]. As reported in previous studies, high adherence to dietary recommendations is associated with improved nutritional and clinical outcomes [28,29]. Therefore, the findings of this study provide a guideline for future nutritional recommendations, emphasizing the need to increase *n*-3 consumption through the diet, complemented with EPA and DHA supplementation at an adequate dose of 1.5 to 3 g/day (with higher doses for carriers of the risk genotype). This approach reinforces the strategy of directly providing *n*-3 products, which are synthesized after the desaturation step affected by the *FADS1* rs174547 variant, as the primary function impaired by this variant is the reduced enzymatic activity of D5D.

Several authors have emphasized the importance of supplementation, recognizing the difficulty in meeting *n*-3 requirements through food alone, particularly due to the variable quality of *n*-3 sources. To establish a precise dosage, further research is needed [30,31]. However, existing studies recommend daily doses ranging from 0.8 g to 2.5 g for adults, with the potential for higher doses in specific pathologies. This may be especially relevant for CC genotype carriers (risk genotype), where increased supplementation could generate a protective effect and help prevent metabolic alterations [26,32]. Moreover, ensuring adherence to dietary recommendations is crucial for achieving positive health and nutritional outcomes, highlighting the significance of promoting compliance with supplementation guidelines.

There were several limitations to this study that should be addressed. First, the sample size was relatively small. Second, adherence to taking the capsules was not documented, as some participants forgot to return their capsule bottle. However, the study had several strengths, including a semi-controlled nutritional intervention and the use of red blood cell fatty acid profiling, which is a more reliable methodology for reflecting long-term fatty acid consumption. The intervention duration was also sufficient to detect differences in fatty acid composition between the groups. Importantly, this is the first study to compare the effects of an *n*-3-supplemented diet on fatty acid composition in RBCs in obese individuals carrying the rs174547 variant within the Mexican population.

Regarding perspectives for future research, based on the results obtained in this study, it is proposed to test different doses of *n*-3 supplementation in subjects carrying the CC genotype of the rs174547 variant. Additionally, it would be valuable to analyze other genetic variants in linkage disequilibrium that may be associated with a dose–response effect. Finally, this study could pave the way for future in vitro research to analyze desaturase activity and further elucidate the molecular mechanism of this variant.

## 5. Conclusions

The effect of the *FADS1* rs174547 variant in subjects with the CC genotype showed minor increases in PUFA percentages and the O3I in the RBCs of individuals with obesity. The O3I confirmed an increase in *n*-3 fatty acids in RBCs following the dietary intervention combined with *n*-3 supplementation, highlighting the importance of dietary intake of *n*-3 fatty acids.

## Figures and Tables

**Table 1:** Allelic and genotypic frequencies of the *FADS1* rs174547 variant.

	Allelic Frequency, *n* (%)		Genotypic Frequency, *n* (%)		
	C	T	CC	CT	TT
Reported Frequency (1000 genomes, AMR)	408 (59)	286 (41)	130 (37.5)	148 (42.7)	69 (19.8)
Reference population (*n* = 314)	179 (57.1)	135 (42.9)	102 (32.5)	155 (49.0)	57 (18.5)
Placebo group (*n* = 38)	20 (52.63)	18 (47.36)	12 (31.58)	16 (42.10)	10 (26.32)
Omega-3 group (*n* = 38)	20 (52.63)	18 (47.36)	10 (26.31)	20 (52.63)	8 (21.05)

Hardy–Weinberg equilibrium (*p* = 0.88). Quantitative variables are expressed in frequency and percentage. CC: wild homozygous; CT: heterozygous; TT: risk homozygous; AMR: admixed American. Chi-square was used.

**Table 2:** Baseline sociodemographic, dietary, and anthropometric data of the study population.

Variable	Placebo Group (*n* = 38)	Omega-3 Group (*n* = 38)	*p*
Demographics			
Age (years)	38.1 ± 11.2	37.1 ± 5.8	0.72
Gender: Female, *n* (%) Male, *n* (%)	26 (68.4) 12 (31.6)	26 (68.4) 12 (31.6)	0.79
Dietary variables			
Energy (Kcal)	2639.4 ± 1125.3	2470.1 ± 525.1	0.97
PT (%)	17.4 ± 4.1	17.3 ± 5.0	0.43
HC (%)	40.6 ± 8.3	46.2 ± 10.4	0.72
LP (%)	42.5 ± 7.9	37.9 ± 8.9	0.60
Cholesterol (mg/day)	577.1 ± 231.1	389.4 ± 239.7	0.61
SFA (%)	15.4 ± 4.4	12.6 ± 3.7	0.29
MUFA (%)	15.5 ± 4.3	12.9 ± 3.5	0.43
PUFA (%)	6.5 ± 3.0	7.4 ± 3.5	0.81
LA 18:2 (g)	17.2 ± 7.8	20.4 ± 13.6	0.49
ALA 18:3 (g)	1.3 ± 0.7	1.5 ± 0.5	0.79
ETA 18:4 (g)	0.001 ± 0.001	0.003 ± 0.004	0.84
AA 20:4 (g)	0.2 ± 0.1	0.1 ± 0.1	0.35
EPA 20:5 (g)	0.005 ± 0.004	0.003 ± 0.05	0.69
DPA 22:5 (g)	0.005 ± 0.003	0.01 ± 0.05	0.96
DHA 22:6 (g)	0.05 ± 0.02	0.06 ± 0.05	0.49

Results were obtained from the dietary records. Qualitative variables are expressed in frequency and percentage, while quantitative variables are expressed in mean and standard deviation. F: female gender; M: male gender; PT: proteins; HC: carbohydrates; LP: lipids; SFA: saturated fatty acid; MUFA: monounsaturated fatty acid; PUFA: polyunsaturated fatty acid; LA: linoleic acid; ALA: alpha linolenic acid; ETA: eicosatetraenoic acid; AA: arachidonic acid; EPA: eicosapentaenoic acid; DPA: docosapentaenoic acid; DHA: docosahexaenoic acid. *t*-test for independent groups was used.

**Table 3:** Baseline biochemical characteristics of the study population.

Variable	Placebo Group (*n* = 38)	Omega-3 Group (*n* = 38)	*p*
Biochemical variables			
Glucose (mg/dL)	94.05 ± 9.4	92.7 ± 9.02	0.65
Triglycerides (mg/dL)	186.5 ± 132.4	165.5 ± 84.7	0.54
Total cholesterol (mg/dL)	190.7 ± 45.8	198.8 ± 35.6	0.53
HDL-c (mg/dL)	37.1 ± 7.6	43.2 ± 14.1	0.09
LDL-c (mg/dL)	116.3 ± 34.6	122.4 ± 30.6	0.55
VLDL-c (mg/dL)	34.5 ± 20.6	33.3 ± 16.8	0.84
Insulin (µU/dL)	33.01 ± 3.6	31.5 ± 4.4	0.85

Quantitative variables are expressed as mean and standard deviation. HDL-c: high-density lipoprotein cholesterol; LDL-c: low-density lipoprotein cholesterol; VLDL-c: very-low-density lipoprotein cholesterol. *t*-test for independent groups was used.

**Table 4:** Changes in dietary variables in both groups.

Nutritional Variables	Placebo Group (*n* = 38)				Omega-3 Group (*n* = 38)				
	Baseline	Final	∆	*p* ^1^	Baseline	Final	∆	*p* ^2^	*p* ^3^
Energy (kcal)	2639.4 ± 1125.3	1809.2 ± 471.5	−830.2 ± 981.1	0.131	2470.1 ± 525.1	1677.4 ± 207.4	−792.6 ± 491.2	0.023	0.941
PT (g)	115.3 ± 58.3	80.2 ± 12.4	−35.1 ± 50.5	0.225	107.3 ± 83.7	83.7 ± 10.9	−23.5 ± 33.09	0.142	0.917
PT (%)	17.4 ± 4.1	18.3 ± 3.5	0.9 ± 2.3	0.500	17.3 ± 5.0	20.0 ± 2.2	2.8 ± 5.1	0.225	0.480
HC (g)	267.9 ± 136.7	185.8 ± 44.2	−82.0 ± 100.5	0.142	285.7 ± 37.2	196.7 ± 31.9	−88.9 ± 65.3	0.038	0.901
HC (%)	40.6 ± 8.3	41.4 ± 7.1	0.7 ± 9.9	0.686	46.2 ± 10.4	46.8 ± 3.0	−1.5 ± 15.5	0.893	0.787
LP (g)	124.9 ± 62.1	88.4 ± 37.02	−36.5 ± 61.9	0.295	104.07 ± 48.2	66.0 ± 13.0	−38.07 ± 54.7	0.261	0.967
LP (%)	42.5 ± 7.9	43.0 ± 7.2	0.3 ± 10.6	0.893	37.9 ± 8.9	35.4 ± 5.3	−0.7 ± 15.2	0.893	0.896
Cholesterol (mg)	577.1 ± 231.1	261.9 ± 111.4	−315.2 ± 334.9	0.170	389.4 ± 239.7	326.3 ± 114.6	−63.08 ± 314.4	0.500	0.255
SFA (g)	45.4 ± 21.9	25.5 ± 7.9	−19.8 ± 20.4	0.095	34.8 ± 20.02	20.1 ± 3.4	−14.7 ± 17.6	0.136	0.680
SFA (%)	15.4 ± 4.4	12.7 ± 2.2	−2.7 ± 3.6	0.138	12.6 ± 3.7	10.8 ± 1.7	−1.07 ± 5.2	0.500	0.569
Lauric acid (g)	0.5 ± 0.2	0.5 ± 0.2	−0.03 ± 0.4	0.902	0.5 ± 0.3	0.3 ± 0.1	−0.1 ± 0.3	0.225	0.575
Palmitic acid (g)	24.9 ± 11.9	15.7 ± 5.1	−9.22 ± 10.6	0.209	20.8 ± 11.7	11.7 ± 1.8	−9.0 ± 11.2	0.248	0.983
Stearic acid (g)	12.7 ± 7.3	4.8 ± 1.4	−7.8 ± 7.0	0.066	8.7 ± 5.8	4.4 ± 1.0	−4.3 ± 5.2	0.134	0.396
MUFA (g)	45.5 ± 311.1	36.4 ± 23.8	−9.1 ± 34.0	0.765	35.5 ± 17.9	24.4 ± 7.5	−11.1 ± 22.9	0.438	0.913
MUFA (%)	15.5 ± 4.3	17.1 ± 6.1	2.5 ± 7.3	0.490	12.9 ± 3.5	13.1 ± 3.6	0.7 ± 7.2	0.043	0.720
Oleic acid (g)	39.09 ± 25.7	34.4 ± 23.1	−4.6 ± 28.2	0.925	31.1 ± 14.09	21.55 ± 7.7	−9.5 ± 20.09	0.421	0.759
PUFA (g)	19.09 ± 8.4	17.9 ± 5.09	−1.1 ± 7.2	0.817	22.4 ± 14.0	13.4 ± 6.5	−9.06 ± 17.5	0.345	0.377
PUFA (%)	6.5 ± 3.0	8.9 ± 0.8	2.2 ± 2.6	0.138	7.4 ± 3.5	6.5 ± 3.0	−0.9 ± 5.3	0.893	0.273
LA 18:2 (g)	17.2 ± 7.8	15.9 ± 4.5	−1.2 ± 6.7	0.838	20.4 ± 13.6	11.3 ± 5.2	−9.07 ± 16.3	0.225	0.354
ALA 18:3 (g)	1.3 ± 0.7	1.3 ± 0.5	−0.04 ± 0.8	0.902	1.5 ± 0.5	1.1 ± 0.5	−0.4 ± 0.8	0.290	0.457
ETA 18:4 (g)	0.001 ± 0.001	0.02 ± 0.01	0.02 ± 0.01	0.099	0.003 ± 0.004	0.007 ± 0.006	0.003 ± 0.008	0.500	0.047
AA 20:4 (g)	0.2 ± 0.1	0.1 ± 0.03	−0.1 ± 0.1	0.104	0.1 ± 0.1	0.1 ± 0.07	−0.008 ± 0.1	0.893	0.212
EPA 20:5 (g)	0.005 ± 0.004	0.1 ± 0.1	0.1 ± 0.1	0.043	0.003 ± 0.05	0.033 ± 0.06	0.03 ± 0.07	0.345	0.076
DPA 22:5 (g)	0.005 ± 0.003	0.1 ± 0.1	0.01 ± 0.02	0.077	0.01 ± 0.05	0.02 ± 0.5	0.007 ± 0.01	0.412	0.382
DHA 22:6 (g)	0.05 ± 0.02	0.2 ± 0.1	0.2 ± 0.2	0.043	0.06 ± 0.05	0.1 ± 0.08	0.06 ± 0.1	0.306	0.117
TFA (g)	0.6 ± 0.7	0.1 ± 0.1	−0.4 ± 0.7	0.887	0.8 ± 0.8	0.3 ± 0.2	−0.5 ± 0.7	0.275	0.754
Sugar (g)	101.9 ± 71.8	62.5 ± 28.6	−39.4 ± 44.1	0.116	100.9 ± 38.09	59.3 ± 19.2	−41.6 ± 46.1	0.114	0.941
Fiber (g)	23.3 ± 9.8	27.3 ± 3.7	1.8 ± 9.6	0.407	37.8 ± 8.2	27.9 ± 10.2	3.5 ± 11.4	0.125	0.398
Sodium (mg)	30007.2 ± 1986.5	1846.9 ± 632.2	−1160.2 ± 1519.4	0.158	2166.1 ± 705.08	1827.7 ± 619.1	−338.4 ± 384.6	0.160	0.275
*n*-6:*n*-3 ratio	12:1	10:1	−2:1	0.282	14:1	10:1	−5:1	0.480	

Results were obtained from the dietary records. Data are expressed in media and standard deviation. PT: proteins; HC: carbohydrates; LP: lipids; SFA: saturated fatty acid; MUFA: monounsaturated fatty acid; PUFA: polyunsaturated fatty acid; TFA: trans-fatty acid; Ac: acid; AL: linoleic acid; ALA: alpha linolenic acid; AA: arachidonic acid; ETA: stearidonic acid; EPA: eicosapentaenoic acid; DHA: docosahexanoic acid. *t*-test for two related variables was used. ∆: Subtraction of the final mean value minus the baseline mean value. A value with a + sign indicates an increase at the end of the intervention, and a value with a − sign indicates a decrease at the end of the intervention. The intragroup comparisons (baseline vs. final) were performed with the paired Student’s *t*-test. Delta (∆) comparisons between groups (intergroup) were analyzed with the unpaired Student’s *t*-test. The *p*^1^ value corresponds to the comparison between final and baseline in the placebo group (intragroup). The *p*^2^ value corresponds to the comparison between final and baseline in the omega-3 group (intragroup). The *p*^3^ value corresponds to the comparison between the groups (intergroup: placebo vs. omega-3 group).

**Table 5:** Changes in the O3I in both groups.

	Placebo Group (*n* = 38)				Omega-3 Group (*n* = 38)			
Variable	Baseline	Final	∆	*p*	Baseline	Final	∆	*p*
O3I (%)	4.9 ± 2.0	7.2 ± 1.6	2.3 ± 2.0	0.001	6.1 ± 2.3	8.1 ± 2.3	1.9 ± 2.2	0.001

Results were obtained from the lipid profile of RBCs via gas chromatography. Data are expressed as mean and standard deviation. Paired *t*-test/Wilcoxon test. ∆: Difference between the final mean value and the baseline mean. O3I: percentage of EPA + DHA fatty acids relative to the total proportion of fatty acids in RBC. The *p*-value corresponds to the comparison between baseline and final measurements within each group.

**Table 6:** Comparison of changes in the fatty acid profiles in RBCs between the CC and TT genotypes of *FASD1* (rs174547) at the end of the intervention in both study groups.

	Placebo Group (*n* = 22)									Omega-3 Group (*n* = 18)										
Variable	Baseline CC (*n* = 12)	Final CC (*n* = 12)	∆ CC (*n* = 12)	*p*^1^ value	Baseline TT (*n* = 10)	Final TT (*n* = 10)	∆ TT (*n* = 10)	*p*^2^ Value	*p*^3^ Value	Baseline CC (*n* = 10)	Final CC (*n* = 10)	∆ CC (*n* = 10)	*p*^4^ Value	Baseline TT (*n* = 8)	Final TT (*n* = 8)	∆ TT (*n* = 8)	*p*^5^ Value	*p*^6^ Value	*p*^7^ Value	*p*^8^ Value
SFA (%)	35.7 ± 3.7	38.3 ± 3.3	2.6 ± 3.4	0.004	36.7 ± 5.1	43.3 ± 9.5	6.5 ± 12.0	0.119	0.796	38.2 ± 5.3	37.0 ± 3.7	−1.1 ± 4.2	0.401	35.9 ± 2.1	40.3 ± 2.7	4.3 ± 1.5	<0.001	0.001	0.015	0.579
Palmitic acid (%)	19.4 ± 2.4	21.1 ± 1.6	1.6 ± 1.7	0.001	18.6 ± 3.2	22.0 ± 4.4	3.3 ± 5.0	0.063	0.524	20.6 ± 2.6	20.5 ± 2.3	−0.02 ± 2.7	0.980	19.4 ± 1.8	21.9 ± 2.2	2.4 ± 0.5	<0.001	0.034	0.101	0.582
Stearic acid (%)	14.9 ± 1.5	15.5 ± 1.4	0.6 ± 1.6	0.106	16.3 ± 2.3	19.0 ± 3.9	2.7 ± 5.5	0.156	0.524	16.4 ± 2.6	14.8 ± 1.4	−1.5 ± 2.1	0.043	15.4 ± 1.1	17.0 ± 0.4	1.6 ± 1.0	0.003	0.001	0.005	0.579
MUFA (%)	35.4 ± 9.1	29.4 ± 8.5	−0.6 ± 11.5	0.041	33.3 ± 8.8	25.6 ± 7.7	0.9 ± 1.9	0.103	0.654	31.1 ± 8.6	34.4 ± 8.4	3.2 ± 8.7	0.265	38.0 ± 5.1	24.4 ± 3.6	−13.5 ± 3.5	<0.001	0.001	0.036	0.209
Oleic acid (%)	13.2 ± 2.4	14.0 ± 1.1	0.7 ± 2.1	0.132	11.2 ± 2.7	12.1 ± 1.0	1.3 ± 1.8	0.157	0.840	13.3 ± 1.1	15.0 ± 6.4	1.7 ± 6.4	0.406	13.0 ± 0.4	12.7 ± 1.6	−0.3 ± 1.9	0.630	0.762	0.650	0.177
PUFA (%)	28.8 ± 7.1	32.1 ± 5.6	3.3 ± 9.4	0.149	29.8 ± 6.4	30.9 ± 8.1	1.1 ± 8.9	0.712	0.538	30.5 ± 3.6	28.4 ± 5.7	−2.1 ± 5.0	0.220	25.9 ± 3.1	35.1 ± 1.6	9.2 ± 3.4	<0.001	0.001	0.056	0.021
*n*-6 (%)	16.4 ± 5.5	18.1 ± 3.8	1.6 ± 5.5	0.211	16.9 ± 4.0	13.7 ± 3.5	3.2 ± 5.2	0.079	0.057	18.3 ± 2.7	14.1 ± 4.8	−4.2 ± 4.9	0.023	14.1 ± 2.1	18.1 ± 1.3	4.0 ± 0.9	<0.001	0.001	0.009	0.001
LA (%)	9.0 ± 3.5	9.3 ± 2.3	0.3 ± 3.7	0.739	9.5 ± 5.3	6.2 ± 1.2	−3.2 ± 5.6	0.097	0.160	9.2 ± 1.4	7.4 ± 2.1	−1.8 ± 1.5	0.005	6.2 ± 1.4	8.0 ± 1.0	1.7 ± 1.2	0.006	0.001	0.099	0.021
AA (%)	7.4 ± 2.4	8.8 ± 1.7	1.3 ± 2.7	0.048	7.3 ± 3.1	7.4 ± 2.3	0.01 ± 3.1	0.986	0.241	9.0 ± 1.6	6.7 ± 2.7	−2.3 ± 3.5	0.060	7.8 ± 1.0	10.3 ± 1.4	2.5 ± 1.0	<0.001	0.012	0.004	0.037
*n*-3 (%)	12.3 ± 3.6	14.0 ± 3.2	1.6 ± 6.3	0.281	12.8 ± 3.6	17.2 ± 5.1	4.3 ± 5.3	0.030	0.272	12.2 ± 2.2	14.3 ± 1.1	2.1 ± 1.7	0.004	11.9 ± 1.1	16.8 ± 1.6	4.9 ± 2.6	0.001	0.026	0.774	0.765
ALA (%)	5.3 ± 3.8	3.0 ± 2.9	−2.2 ± 5.3	0.089	2.4 ± 3.4	0.6 ± 0.5	−1.7 ± 3.0	0.100	0.790	3.4 ± 2.2	3.7 ± 2.4	0.3 ± 3.2	0.738	3.6 ± 1.7	5.5 ± 2.8	1.9 ± 4.4	0.264	0.762	0.171	0.054
EPA (%)	1.5 ± 0.3	1.5 ± 0.2	0.02 ± 0.4	0.724	1.1 ± 0.6	1.5 ± 0.2	0.4 ± 0.3	0.006	0.009	1.7 ± 0.2	1.4 ± 0.3	−0.2 ± 0.4	0.152	1.5 ± 0.07	1.9 ± 1.2	0.4 ± 0.1	<0.001	0.003	0.093	0.966
DPA (%)	0.9 ± 0.3	0.9 ± 0.1	0.001 ± 0.3	0.988	2.0 ± 1.7	0.9 ± 0.3	−1.1 ± 1.8	0.094	0.408	1.4 ± 0.9	1.5 ± 0.5	0.02 ± 1.3	0.951	0.9 ± 0.1	1.5 ± 0.3	0.6 ± 0.2	<0.001	0.200	0.954	0.018
DHA (%)	3.7 ± 2.0	5.2 ± 1.3	1.49 ± 1.7	0.002	2.0 ± 0.7	5.3 ± 1.2	3.2 ± 2.0	0.001	0.024	5.3 ± 2.1	6.3 ± 2.2	0.9 ± 2.5	0.254	4.9 ± 1.0	7.7 ± 2.1	2.7 ± 2.2	0.010	0.284	0.583	0.630
O3I (%)	5.3 ± 2.2	6.8 ± 1.5	1.51 ± 1.8	0.003	3.2 ± 0.5	6.8 ± 1.4	3.62 ± 1.7	<0.001	0.007	7.0 ± 2.0	7.8 ± 2.5	0.7 ± 2.7	0.403	6.5 ± 1.0	9.6 ± 2.1	3.1 ± 2.2	0.006	0.064	0.438	0.607

Results were obtained from the lipid profile of RBCs via gas chromatography. Data are expressed as mean and standard deviation. SFA: saturated fatty acid; MUFA: monounsaturated fatty acid; AL: linoleic acid; AA: arachidonic acid; ALA: alpha linolenic acid; EPA: eicosapentanoic acid; DPA: docosapentanoic acid; DHA: docosahexanoic acid; PUFA: polyunsaturated fatty acid. Independent *t*-test/U of Mann–Whitney test. ∆: Difference between the final mean value and the baseline mean. A value with a − sign indicates a decrease at the end of the intervention. O3I: percentage of EPA + DHA fatty acids relative to the total proportion of fatty acids in RBC. *p*^1^ corresponds to the comparison between final and baseline in CC genotype carriers in the placebo group. *p*^2^ corresponds to the comparison between final and baseline in TT genotype carriers in the placebo group. *p*^3^ corresponds to the comparison between ∆CC and ∆TT in the placebo group. *p*^4^ corresponds to the comparison between final and baseline in CC genotype carriers in the omega-3 group. *p*^5^ corresponds to the comparison between final and baseline in TT genotype carriers in the omega-3 group. *p*^6^ corresponds to the comparison between ∆CC and ∆TT in the omega-3 group. *p*^7^ corresponds to the comparison between ∆CC values between the groups (intergroup: ∆CC of placebo vs. ∆CC of omega-3 group). *p*^8^ corresponds to the comparison between ∆TT values between the groups (intergroup: ∆TT of placebo vs. ∆TT of omega-3 group).

## Data Availability

The original contributions presented in the study are included in the article/Supplementary Material, further inquiries can be directed to the corresponding author.

## Supplementary Materials

The following supporting information can be downloaded at: https://www.mdpi.com/article/10.3390/nu16203522/s1, Table S1: Total of fatty acids measured by gas chromatography.
