# Supplementary material for: FADS1 Genetic Variant and Omega-3 Supplementation Are Associated with Changes in Fatty Acid Composition in Red Blood Cells of Subjects with Obesity
Source: Nutrients. 2024 Oct 17;16(20):3522. doi: 10.3390/nu16203522 (PMC11509948; doi:10.3390/nu16203522)
Supplement: Supplementary file 1 [file nutrients-16-03522-s001.zip › nutrients-3202089-supplementary.pdf]

Supplemenntary Material

Table S1. **Total of fatty acids measured by gas chromatography**

Fatty acids measured by gas chromatography				
**Saturated**	**Monounsaturated**	**n-9 PUFA**	**n-6 PUFA**	**n-3 PUFA**
10:0	16:1n-9	18:2n-9	18:2n-6	16:4n-3
12:0	16:1n-7	20:2n-9	18:3n-6	18:3n-3
14:0	18:1n-9	20:3n-9	20:2n-6	18:4n-3
15:0	18:1n-7	C22n-9	20:3n-6	20:3n-3
16:0	20:1n-7		20:4n-6	20:4n-3
18:0	20:1n-9		22:4n-6	20:5n-3
20:0	20:1n-11		22:5n-6	22:4n-3
22:0	22:1n-9			22:5n-3
	24:1n-9			22:6n-3
